# Falcipain 2 inhibitors and antiplasmodial compounds from a bio-guided fractionation of the fruits of *Sorindeia juglandifolia* A. Rich. (Anacardiaceae) growing in Cameroon

**DOI:** 10.1186/1475-2875-9-S2-P6

**Published:** 2010-10-20

**Authors:** Fabrice F Boyom, Eugénie K Madiesse, Jean J Bankeu, Valere P Tsouh, Bruno N Lenta, Wilfred F Mbacham, Etienne Tsamo, Paul HA Zollo, Jiri Gut, Philip J Rosenthal

**Affiliations:** 1 Laboratory of Phytobiochemistry, Department of Biochemistry, Faculty of Science, University of Yaoundé 1, P.O., Box 812, Yaoundé, Cameroon; 2 Laboratory for Public Health Biothechnology, Biotechnology Centre, UniversityofYaoundé 1, P.O. Box 8094, Yaoundé, Cameroon; 3 Laboratory of Natural Products, Department of Organic Chemistry, Faculty of Science, University of Yaoundé 1, P.O. Box 812, Yaoundé, Cameroon; 4 Division of Infectious Diseases, Department of Medicine, University of California; San Francisco, 94943 USA

## Background

Discovering new lead compounds with the potential to become usable drugs against malaria is a crucial step to ensuring a sustainable global pipeline for innovative products. We describe here the results of an antimalarial activity-driven fractionation of the fruits *of Sorindeia juglandifolia* growing in Cameroon.

## Materials and methods

Fresh fruits were collected by an ethnobotanist in Yaoundé area in May 2009. The plant was dried at Room Temperature during 7 days, powdered and extracted using organic solvents. The extract was fractionated by flash chromatography over silica gel (70-230 mesh, Merck, 7 x 42 cm), eluting with gradients of hexane-ethyl acetate mixtures, and resulted in 35 fractions, which were pooled on the basis of thin layer chromatography patterns. Resulting fractions were tested *in vitro* against the *Plasmodium falciparum* chloroquine-resistant strain W2, and the recombinant cysteine protease Falcipain 2 (F2) [1]. Two fractions showed the best potency and were selected for phytochemical investigation guided by biological activity.

## Results

**Figure 1:**
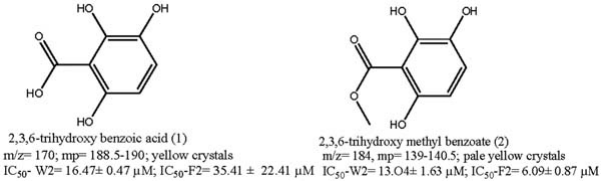
Antimalarial compounds isolated from *Sorindeia juglandifolia*.

The main end-compounds afforded through the phytochemical investigation were found to be known (Figure 1), 2,3,6-trihydroxy benzoic acid (1), and 2,3,6-trihydroxy methyl benzoate (2) that exhibited low micromolar inhibitory concentrations against *P. falciparum* W2 and Falcipain 2 respectively.

## Conclusion

The isolated compounds have not been previously investigated for antimalarial activity, and therefore suggesting further investigation.
